# Association of sleep symptom burden with default mode network homogeneity in first-episode drug-naïve adolescents with major depressive disorder

**DOI:** 10.3389/fpsyt.2026.1886224

**Published:** 2026-08-06

**Authors:** Yixiang You, Lili Meng, Shan Li, Zongze Li, Changyong Zhang, Yulin Cao

**Affiliations:** 1 School of Medicine, Wuhan University of Science and Technology, Wuhan, China; 2 Department of Psychiatry, Wuhan Wudong Hospital, Wuhan, China; 3 Department of Psychiatry, Wuhan Mental Health Center, Wuhan, China; 4 Department of Psychiatry, Wuhan Hospital for Psychotherapy, Wuhan, China

**Keywords:** adolescents, default mode network, major depressive disorder, network homogeneity, resting-state fMRI, sleep symptom burden

## Abstract

**Background:**

The default mode network (DMN) plays an important role in depression and sleep regulation. However, whether sleep symptoms are associated with alterations in DMN homogeneity in adolescent major depressive disorder (MDD) remains unclear. This study aimed to investigate whether sleep symptom burden is associated with alterations in DMN homogeneity in adolescents with MDD.

**Methods:**

Following quality control procedures, the final sample comprised 162 first-episode, drug-naïve adolescents with MDD, including 86 participants with higher sleep symptom burden (MDD-S) and 76 participants with lower sleep symptom burden (MDD-NS), together with 53 healthy controls (HCs). Sleep grouping was based on Hamilton Depression Rating Scale (HAMD) sleep items. The DMN was identified using group independent component analysis (ICA). Voxel-wise network homogeneity (NH) values within the DMN were calculated and compared among groups. Correlation analyses and exploratory support vector machine (SVM) analyses were performed.

**Results:**

Opposite-direction NH alterations in the precuneus were observed between groups with different sleep symptom burdens. Compared with HCs, the MDD-NS group showed increased NH in the right precuneus, whereas the MDD-S group exhibited decreased NH in the bilateral precuneus. Relative to the MDD-NS group, the MDD-S group showed increased NH in the left anterior cingulate cortex, posterior cingulate cortex, and angular gyrus, together with decreased NH in the right middle temporal gyrus. Exploratory SVM analyses showed modest discriminative performance, indicating limited utility of NH alterations alone for individual-level classification.

**Conclusion:**

Adolescents with MDD and different levels of sleep symptoms exhibit distinct DMN homogeneity patterns. Sleep symptoms may be associated with altered DMN synchronization patterns, particularly within the precuneus. The opposite-direction precuneus alterations observed across MDD subgroups may provide a potential perspective for understanding some of the heterogeneous DMN findings reported in previous depression studies.

## 1 Introduction

Major depressive disorder (MDD) is a highly prevalent psychiatric disorder affecting more than 280 million people worldwide (1). Sleep disturbances are among the most common comorbid symptoms of depression, with approximately 80% – 90% of patients experiencing sleep disturbances, including difficulty initiating or maintaining sleep, and early morning awakening (2, 3). Importantly, the relationship between sleep disturbances and depression is bidirectional: sleep disturbances not only increase the risk of developing depression by two- to three-fold, but also contribute to symptom persistence and relapse (4). Clinically, patients with MDD accompanied by sleep disturbances tend to exhibit more severe symptoms, poorer treatment response, and higher relapse rates (5). These findings suggest that sleep disturbances may contribute to clinical and neurobiological heterogeneity in MDD.

Resting-state functional magnetic resonance imaging (rs-fMRI) has been widely used to investigate brain network dysfunction in MDD. Among large-scale brain networks, the default mode network (DMN), which is critically involved in self-referential processing and emotional regulation, has received considerable attention (6). Previous studies using various imaging metrics, including functional connectivity (FC), amplitude of low-frequency fluctuation (ALFF), and regional homogeneity (ReHo), have consistently reported abnormalities within key DMN regions such as the precuneus, posterior cingulate cortex, and medial prefrontal cortex (7–10). However, the direction of these alterations remains inconsistent, which has hindered a unified understanding of DMN dysfunction in MDD. Some studies have reported increased DMN connectivity, which has been interpreted as excessive self-focus and rumination (11), whereas others have found decreased connectivity, suggesting impaired network integration (12). These inconsistencies may be related to differences in sample characteristics, analytical methods, and insufficient consideration of symptom-related heterogeneity.

In the context of depression with sleep disturbances, rs-fMRI findings are similarly heterogeneous. Several studies have reported reduced functional connectivity within the DMN, particularly in core regions such as the precuneus and posterior cingulate cortex (13, 14), which has been associated with poor sleep quality. Comparable reductions in functional connectivity and metabolic activity have also been observed in individuals with primary sleep disturbances (15). In contrast, other studies have identified increased local activity or enhanced synchrony in specific DMN regions, such as the anterior cingulate cortex and angular gyrus, which have been interpreted as compensatory responses to sleep deprivation and emotional stress (16, 17). Furthermore, imaging studies of primary insomnia have also demonstrated reduced functional connectivity and metabolic activity in the precuneus and temporal regions (18, 19). One possible explanation for the inconsistent DMN findings in depression studies is that sleep symptom burden has not been adequately considered. These conflicting findings suggest that sleep disturbances may exert complex effects on DMN function, potentially influencing both large-scale network integration and local synchronization patterns.

Despite these advances, several important issues remain unresolved. First, most existing studies have focused on seed-based functional connectivity, which primarily reflects long-range inter-regional communication, whereas the overall functional integration within the network has received less attention. Network homogeneity (NH) provides a data-driven measure of voxel-wise synchronization within a network without requiring predefined seed regions (20). NH has been increasingly applied in psychiatric research and has demonstrated high sensitivity in detecting network-level abnormalities (21, 22). However, the application of NH in examining sleep-related heterogeneity in depression remains limited. Second, the number of imaging studies directly comparing patients with different levels of sleep symptoms remains limited, and the results are inconsistent. For example, some studies suggest weakened DMN functional connectivity in patients with sleep disturbances (13), while others have found no significant differences or observed localized enhancements (17, 23). Furthermore, previous studies have primarily focused on adult samples, whereas adolescence is a critical stage for brain network development and plasticity (24), and the DMN may exhibit different response patterns to sleep disruption compared to adults (25). Therefore, systematically comparing sleep-related differences in DMN functional integration in an adolescent sample is of great significance for elucidating their underlying neurobiological mechanisms. In addition, previous imaging studies have mainly focused on identifying regional abnormalities, whereas the potential contribution of sleep symptoms to inconsistent DMN findings in depression has received relatively limited attention. Investigating sleep-related alterations in network synchronization may therefore provide additional insight into the neurobiological heterogeneity of adolescent depression.

To address these gaps, the present study investigated DMN homogeneity in treatment-naïve adolescents with first-episode MDD categorized according to sleep symptom burden. The study aimed to (1) identify sleep-related alterations in DMN homogeneity and (2) characterize differences in DMN synchronization patterns associated with sleep symptoms. We hypothesized that adolescents with MDD and different levels of sleep symptoms would exhibit distinct NH patterns within key DMN regions, reflecting sleep-related neurobiological heterogeneity. To our knowledge, no previous study has directly compared DMN homogeneity according to sleep symptom burden in adolescents with MDD.

## 2 Materials and methods

### 2.1 Participants

From February 2025 to January 2026, right-handed adolescents with first-episode major depressive disorder (MDD) were recruited from the inpatient and outpatient departments of the Wuhan Mental Health Center. Inclusion criteria: (1) Met the diagnostic criteria for major depressive disorder in the Diagnostic and Statistical Manual of Mental Disorders, 5th Edition (DSM-5), as independently confirmed by two psychiatrists; (2) First episode of major depressive disorder, as assessed by the 17-item Hamilton Depression Rating Scale (HAMD-17) (26), with a total score ≥ 17 and a score of ≥ 2 on item 1; (3) Duration of illness < 12 months; (4) No history of psychotropic medication use or electroconvulsive therapy; (5) Age 11–18 years. Exclusion criteria: (1) Coexisting psychiatric or neurological disorders; (2) Pregnancy, history of substance abuse, or severe somatic illness; (3) Initial MRI scan showing structural brain abnormalities; (4) Development of psychotic features or history of bipolar disorder.

Patients were categorized according to sleep symptom burden using the HAMD-17 sleep factor score, calculated as the sum of the three sleep-related items of the HAMD-17. Consistent with previous clinical and neuroimaging studies, a sleep factor score ≥4 was defined as a higher sleep symptom burden, whereas a score ≤3 was defined as a lower sleep symptom burden. Accordingly, participants were assigned to the MDD with higher sleep symptom burden group (MDD-S) or the MDD with lower sleep symptom burden group (MDD-NS), respectively. This cutoff has been widely used to distinguish patients with relatively high versus low insomnia symptom severity and has demonstrated clinical utility in characterizing sleep-related heterogeneity in depression (2, 27). These labels reflect differences in sleep symptom burden rather than a formal diagnosis of insomnia disorder. Ultimately, 86 patients were included in the MDD-S group and 76 in the MDD-NS group. Concurrently, 53 age- and gender-matched healthy controls (HCs) were recruited from the community. The HCs had no history of mental illness, substance abuse, or neurological disorders, and no family history of mental illness. No dedicated sleep questionnaires or objective sleep assessments were administered to the healthy control group. After diagnostic confirmation, MRI quality control, and application of all exclusion criteria, the final sample consisted of 86 adolescents in the MDD-S group, 76 adolescents in the MDD-NS group, and 53 healthy controls.

This study was approved by the Medical Research Ethics Committee of the Wuhan Mental Health Center (Approval No.: KY2025.1119.02). All participants and their legal guardians signed written informed consent forms.

### 2.2 Acquisition and preprocessing of imaging data, and NH calculation

Resting-state functional MRI data were acquired using an Ingenia 3.0T MRI scanner at the Wuhan Mental Health Center. During the scan, participants lay supine with their eyes closed and remained awake; soft earplugs and foam pads were used to minimize head motion and scanning noise. The imaging parameters were as follows: repetition time (TR) = 2500 ms, echo time (TE) = 25 ms, flip angle = 90°, matrix = 64 × 64, slice thickness = 3.5 mm, number of slices = 39, field of view (FOV)=240 × 240 mm², and 200 volumes were collected.

Data preprocessing was performed using the DPARSF software package (28). To ensure signal balance, the first 10 time points for each subject were discarded. The remaining 190 time points underwent head motion correction and inter-slice temporal alignment. Subjects with head motion exceeding 2 mm in translation or 2° in rotation were excluded. Images were spatially standardized to the Montreal Neurological Institute (MNI) template, and voxel size was resampled to 3 × 3 × 3 mm. The resulting imaging data underwent temporal bandpass filtering (0.01–0.08 Hz) and linear detrending. Several spurious covariates were also removed from the images, including signals from region-of-interest (ROI) regions based on ventricular seeds and white matter centers, as well as 24 head motion parameters obtained via rigid body correction. During preprocessing of resting-state FC data, global signals were retained, as recommended in a previous study (29).

Group independent component analysis (group ICA) was performed using the GIFT toolbox to identify the DMN across all participants. The analysis steps included data dimensionality reduction, independent component separation, and reverse reconstruction. Default network components for each subject were selected and extracted based on templates provided by GIFT, and the default network components of all subjects were superimposed to generate a default network mask (28). The spatial map of the ICA-derived DMN component is presented in **Supplementary Figure 1**. The identified component showed substantial spatial overlap with the canonical DMN, supporting the validity of the network definition used for subsequent NH analyses. Network homogeneity (NH) analysis was performed within the DMN in MATLAB. For each participant, the Pearson correlation coefficients were calculated between each voxel within the default network mask and all other voxels within that mask; the average correlation coefficient for that voxel was defined as its NH value. Fisher’s r-to-z transformation was subsequently applied to improve the normality of the correlation coefficients. An average NH map within the DMN was then generated for each participant and smoothed using a 4-mm full-width-at-half-maximum (FWHM) Gaussian kernel (20).

### 2.3 Support vector machine analysis

Support vector machine (SVM) analysis was performed using the LIBSVM V3.1 software package in MATLAB 2013b (MathWorks, USA). A radial basis function (RBF) kernel was applied for classification analysis. Prior to model training, all features were normalized using z-score transformation to minimize scaling effects across variables. A leave-one-out cross-validation (LOOCV) strategy was employed, in which one subject was used as the test sample while the remaining subjects were used for model training in each iteration. Parameter optimization and feature selection were performed exclusively within the training dataset to avoid information leakage. This process was repeated until each participant had served as the test sample once, and the overall classification accuracy was calculated. Given the moderate sample size, LOOCV was adopted as an exploratory validation strategy rather than a clinically generalizable framework (30).

### 2.4 Statistical analysis

Statistical analysis of demographic and clinical data was performed using SPSS 26.0. Voxel-wise NH differences among the three groups were examined using one-way analysis of covariance (ANCOVA), with age, sex, years of education, and mean framewise displacement (FD) included as covariates. Demographic variables included as covariates in the imaging analyses are summarized in **Table 1**. Regions showing significant omnibus group effects were subsequently subjected to *post hoc* pairwise comparisons within the general linear model framework, while retaining the same covariates (age, sex, years of education, and mean FD). Correction for multiple comparisons was performed using Gaussian Random Field (GRF) theory, with a voxel-level threshold of p < 0.001 and a cluster-level threshold of p < 0.05 (two-tailed). Detailed statistical information for significant imaging findings, including omnibus ANCOVA F-values, *post hoc* comparison T-values, cluster sizes, and peak MNI coordinates, is provided in **Tables 2** and **3**. Because head motion may confound resting-state fMRI analyses, mean FD values were calculated for each participant and included as a nuisance covariate, consistent with previous studies (31). For correlation analyses, the normality of clinical variables and extracted NH values was assessed using the Shapiro–Wilk test. Pearson correlation analyses were conducted when both variables were normally distributed; otherwise, Spearman rank correlation analyses were applied. A two-tailed p-value < 0.05 was considered statistically significant.

**Table 1:** Demographic and clinical characteristics of the participants.

Demographic data	MDD-NS group(n=76)	MDD-S group(n=86)	HCs group(n=53)	T (F, or χ2)	P
Age(years)	14.61 ± 1.32	14.85 ± 1.43	15.66 ± 2.05	7.450	<0.001^a^
Years of education	8.62 ± 1.32	8.84 ± 1.43	9.68 ± 2.05	7.583	<0.001^a^
Gender (male/female)	76(19/57)	86(21/65)	53(13/40)	0.008	0.996^b^
HAMD scores	24.49 ± 11.33	27.16 ± 5.92	N/A^d^	1.848	0.067^c^
SLEEP factors scores	2.16 ± 0.91	5.16 ± 0.81	N/A^d^	22.082	<0.001 ^c^
HAMD non-sleep scores	22.33 ± 11.36	22.00 ± 5.69	N/A^d^	-0.228	0.820 ^c^

Values are mean ± SD or n.

HAMD non-sleep score = HAMD total score minus the three HAMD sleep items.

MDD, major depressive disorder; HCs, healthy controls; HAMD, Hamilton Depression Rating Scale; Sleep factor score=Difficulty falling asleep+ Light sleep+ Waking up early; MDD NS, Patients with MDD without SD; MDD-S, Patients with MDD with SD; N/A, Not Applicable.

^a^ANOVA.

^b^Chi-square test.

^c^Two sample t-test.

^d^HAMD scores were not collected for the Healthy Control group, as the HAMD is a clinician-rated scale for assessing depressive symptomatology in patients.

**Table 2:** Brain regions showing significant omnibus ANCOVA group effects in network homogeneity (NH) within the default mode network (DMN).

Brain region	Peak (MNI)			Number of voxels	F-value
	X	y	z		
MDD-S vs. MDD-NS vs. HCs groups					
Right Precuneus	9	-63	30	25	7.98
Left Precuneus	0	-57	42	27	7.10

MNI, Montreal Neurological Institute; MDD, major depressive disorder; HCs, healthy controls; NH, Network Homogeneity.

Results are based on voxel-wise ANCOVA with age, sex, years of education, and mean framewise displacement (FD) included as covariates. Significant clusters were identified using Gaussian Random Field (GRF) correction (voxel-level p < 0.001; cluster-level p < 0.05).

**Table 3:** Results of covariate-adjusted *post hoc* pairwise comparisons of NH values among groups.

Cluster location	Peak (MNI)			Number of voxels	T-value
	X	y	Z		
MDD-S vs. HCs groups					
Right Precuneus	6	-48	18	20	-2.52
Left Precuneus	0	-57	42	46	-3.76
MDD-NS vs. HCs groups					
Right Precuneus	6	-66	27	40	3.44
MDD-S vs. MDD-NS groups					
Left Anterior Cingulum	-3	51	0	114	2.68
Left Posterior Cingulum	-12	-54	27	247	2.89
Left Angular Gyrus	-45	-57	24	105	2.32
Right Middle Temporal Gyrus	48	-63	21	52	-1.48

MNI, Montreal Neurological Institute; MDD, major depressive disorder; HCs, healthy controls; NH, Network Homogeneity.

*Post hoc* pairwise comparisons were performed within the general linear model framework with age, sex, years of education, and mean framewise displacement (FD) retained as covariates. T-values represent covariate-adjusted pairwise contrasts. Significant clusters were identified using Gaussian Random Field (GRF) correction (voxel-level p < 0.001; cluster-level p < 0.05).

## 3 Results

### 3.1 Demographic characteristics and clinical information

**Figure 1:**
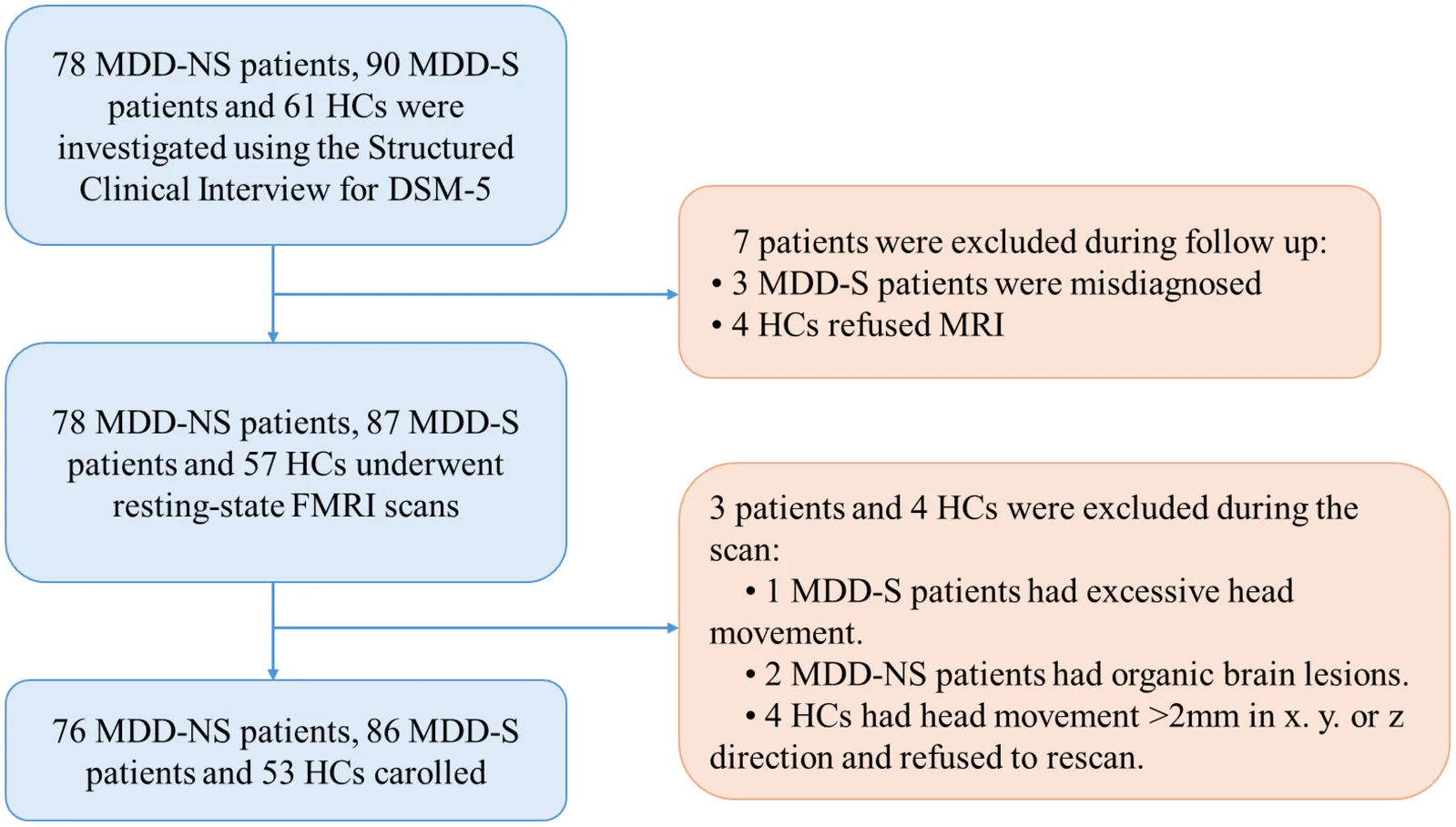
Flow chart of the selection process for participants. MDD, major depressive disorder; HCs, healthy controls; HAMD, Hamilton Depression Rating Scale; Sleep factor score=Difficulty falling asleep+ Light sleep+ Waking up early; MDD NS, Patients with MDD without SD; MDD-S, Patients with MDD with SD.

Fourteen participants were excluded for the following reasons: 3 patients with MDD-S were misdiagnosed during follow-up; 4 healthy controls (HCs) refused MRI for personal reasons; 1 patient with MDD-S exhibited excessive head movement; 2 patients with MDD-NS had organic brain disease; and 4 HCs were excluded because their head movement in the x, y, or z directions exceeded 2 mm and they refused to undergo a repeat scan. The final analysis included 76 participants in the group without sleep disturbances, 86 in the group with sleep disturbances, and 53 healthy controls (**Figure 1**). There was a significant difference in age among the three groups (F = 7.450, p < 0.001) and years of education (F = 7.583, p < 0.001). *Post hoc* analyses indicated that healthy controls were significantly older and had more years of education than both MDD groups (all p < 0.05). Therefore, age and years of education were included as covariates in all voxel-wise NH analyses to minimize potential confounding effects. No significant differences were observed in sex distribution among the three groups (χ² = 0.008, p = 0.996). The HAMD total score was numerically higher in the MDD-S group than in the MDD-NS group; however, this difference did not reach statistical significance (t = 1.848, p = 0.067). As expected, the sleep factor score was significantly higher in the MDD-S group (5.16 ± 0.81) than in the MDD-NS group (2.16 ± 0.91; t = 22.082, p < 0.001) (**Table 1**). To further determine whether subgroup differences were attributable to sleep symptom burden rather than overall depressive severity, we calculated a non-sleep HAMD score by excluding the three sleep items from the total HAMD score. The two MDD groups did not differ significantly on the non-sleep HAMD score (t = −0.228, p = 0.820), suggesting comparable levels of depressive symptom severity apart from sleep-related symptoms.

### 3.2 Differences among NH groups

**Figure 2:**
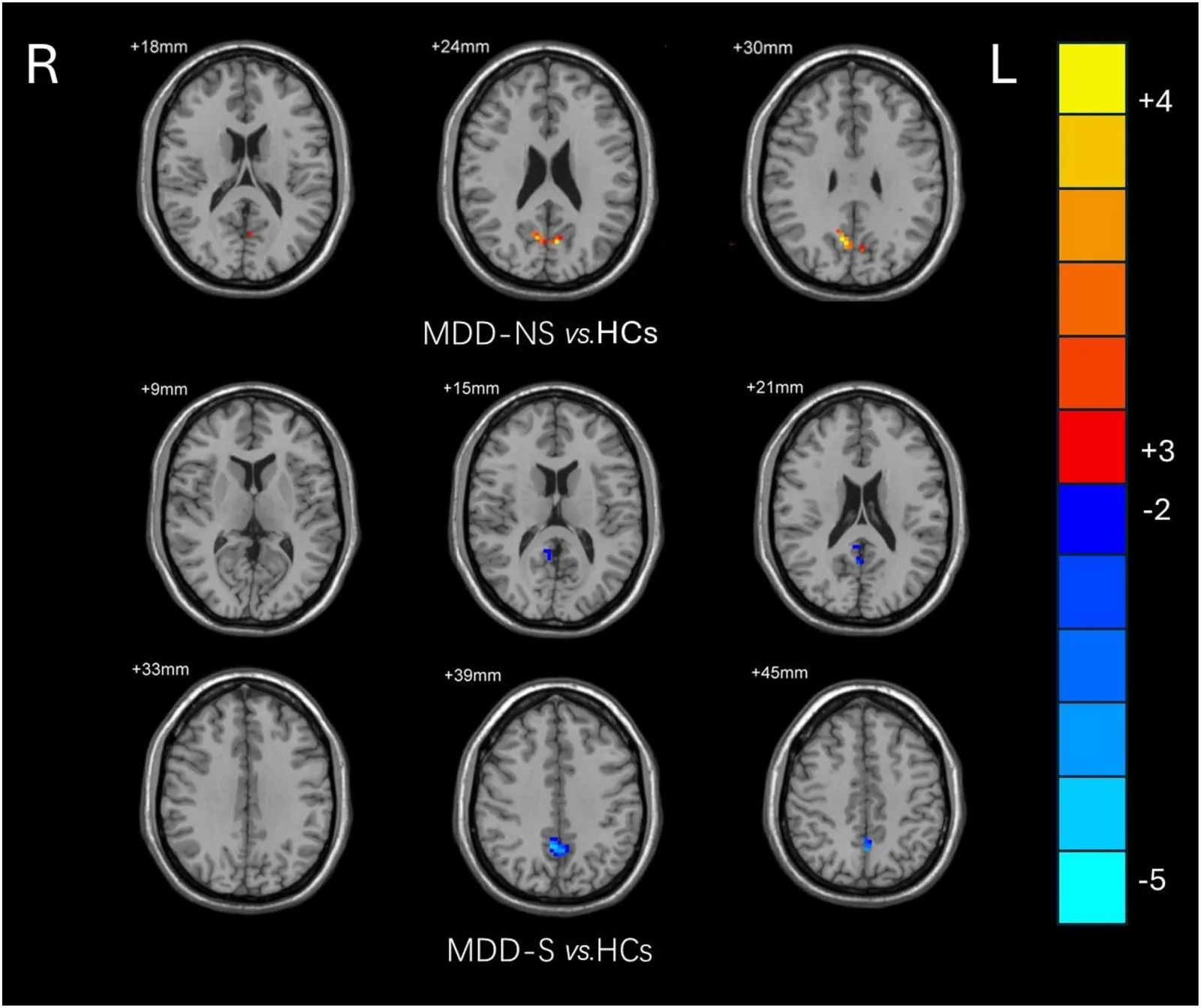
Differences in network homogeneity between MDD-NS vs. HCs and MDD-S vs. HCs. All significant clusters (red: increased NH; blue: decreased NH) are located within the default mode network (DMN). The DMN was identified using group independent component analysis (ICA). The ICA-derived DMN mask is shown in **Supplementary Figure 1**. Statistical maps were thresholded using Gaussian Random Field (GRF) correction with a voxel-level threshold of p < 0.001 and a cluster-level threshold of p < 0.05. Color bar indicates T-values from two-sample t-tests. L, left; R, right. MDD-NS, MDD patients with lower sleep symptom burden; MDD-S, MDD patients with higher sleep symptom burden; HCs, healthy controls.

**Figure 3:**
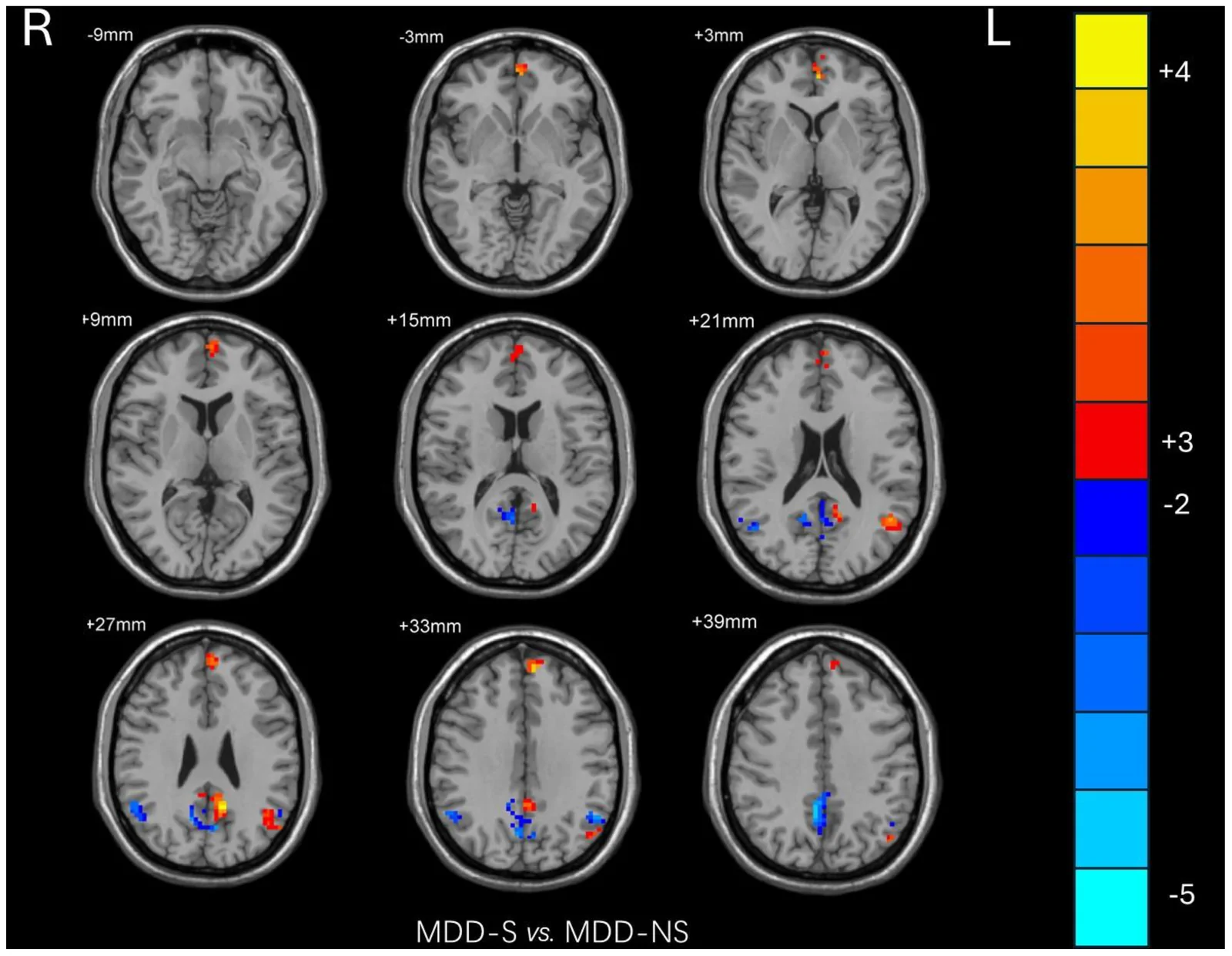
Differences in network homogeneity between MDD-S and MDD-NS. All significant clusters (red: increased NH; blue: decreased NH) are located within the default mode network (DMN). The DMN was identified using group independent component analysis (ICA). The ICA-derived DMN mask is shown in **Supplementary Figure 1**. Statistical maps were thresholded using Gaussian Random Field (GRF) correction with a voxel-level threshold of p < 0.001 and a cluster-level threshold of p < 0.05. Color bar indicates T-values from two-sample t-tests. L, left; R, right. MDD-NS, MDD patients with lower sleep symptom burden; MDD-S, MDD patients with higher sleep symptom burden; HCs, healthy controls.

The ICA-derived DMN component demonstrated substantial spatial overlap with the canonical DMN (**Supplementary Figure 1**), supporting the validity of the network definition used for subsequent NH analyses. A voxel-wise ANCOVA revealed significant group effects in the right precuneus and left precuneus within the DMN (**Table 2**). Subsequent covariate-adjusted pairwise comparisons revealed that the MDD-S group showed significantly decreased NH values in the bilateral precuneus compared with HCs, whereas the MDD-NS group exhibited significantly increased NH values in the right precuneus relative to HCs. Direct comparisons between the two MDD subgroups further revealed significant NH differences in the left anterior cingulate cortex, left posterior cingulate cortex, left angular gyrus, and right middle temporal gyrus (**Table 3**; **Figures 2**, **3**).

MDD-S vs. HCs groups

Compared with the healthy control group, the group with sleep disturbances showed significantly reduced NH in the bilateral precuneus within the DMN (**Table 3**; **Figure 2**).

MDD-NS vs. HCs groups

Compared with the healthy control group, the group without sleep disturbances showed significantly increased NH within the DMN of the right precuneus (**Table 3**; **Figure 2**).

MDD-S vs. MDD-NS groups

Compared with the group without sleep disturbances, the group with sleep disturbances showed significantly increased NH within the DMN in the left anterior cingulate, left posterior cingulate, and left angular gyrus and significantly decreased NH within the DMN in the right middle temporal gyrus, as shown in **Table 2** and **Figure 3** (p < 0.05). (**Table 3**; **Figure 3**).

### 3.3 Correlation between NH values and clinical characteristics

Correlation analyses were performed between NH values extracted from significant clusters and clinical variables, including HAMD total scores and sleep factor scores. No significant correlations were observed between NH values and any clinical variable (all p > 0.05). Detailed correlation coefficients and corresponding p values are presented in **Supplementary Table 1**.

### 3.4 SVM results

**Figure 4:**
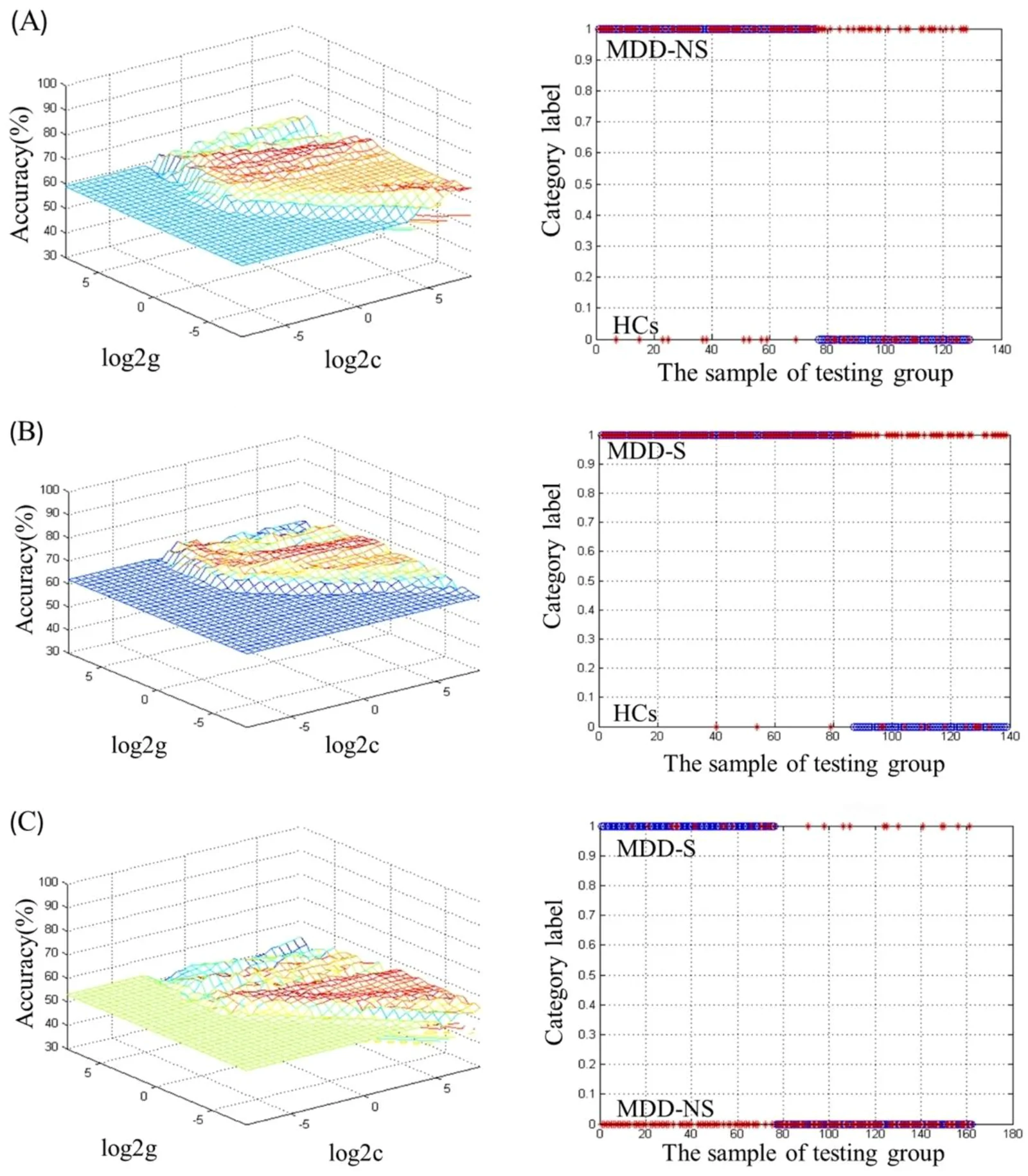
Support vector machine (SVM) classification results based on NH features. **(A)** Classification of MDD-NS and healthy controls using NH values from the right precuneus. The left panel shows the 3D parameter-selection surface obtained during SVM optimization, and the right panel displays the classification results. Each point represents an individual participant. The overall classification accuracy was 68.22%. **(B)** Classification of MDD-S and healthy controls using NH values from the bilateral precuneus. The left panel shows the 3D parameter-selection surface obtained during SVM optimization, and the right panel displays the classification results. Each point represents an individual participant. The overall classification accuracy was 68.35%. **(C)** Classification of MDD-S and MDD-NS using NH values from the left anterior cingulate cortex, left posterior cingulate cortex, left angular gyrus, and right middle temporal gyrus. The left panel shows the 3D parameter-selection surface obtained during SVM optimization, and the right panel displays the classification results. Each point represents an individual participant. The overall classification accuracy was 58.02%. NH, network homogeneity; MDD-S, major depressive disorder with higher sleep symptom burden; MDD-NS, major depressive disorder with lower sleep symptom burden. SVM classification results are exploratory; no statistical correction was applied.

Support vector machine classification analysis was performed using NH values extracted from regions showing group differences. Classification based on NH values in the right precuneus achieved an accuracy of 68.22% in distinguishing MDD-NS from healthy controls. The accuracy of distinguishing MDD-S from healthy controls based on bilateral precuneus NH values was 68.35%. Based on NH values in the left anterior cingulate, left posterior cingulate, left angular gyrus, and right middle temporal gyrus, exploratory subgroup differentiation yielded modest classification performance (58.02%). (**Figure 4**) Due to the exploratory nature of the analysis, only overall classification accuracy was retained; other performance metrics (e.g., sensitivity, specificity, AUC) were not available for retrospective reporting.

## 4 Discussion

In this study, we investigated DMN homogeneity alterations in first-episode, drug-naïve adolescents with MDD, with particular emphasis on the potential influence of sleep disturbances on network synchronization patterns. Specifically, patients with lower sleep symptom burden showed increased NH in the right precuneus, whereas those with higher sleep symptom burden exhibited decreased NH in the same region. In addition, increased NH in the anterior cingulate cortex (ACC), posterior cingulate cortex (PCC), and angular gyrus, as well as reduced NH in the middle temporal gyrus (MTG), were identified in the MDD group with higher sleep symptom burden. Exploratory SVM analyses further suggested that NH alterations contain limited group-level discriminative information. Notably, the non-sleep HAMD scores did not differ significantly between the MDD-S and MDD-NS groups. This finding reduces the likelihood that the observed NH differences were solely attributable to overall depressive symptom severity, while lending additional support to the interpretation that sleep symptom burden contributed to the subgroup differences.

A key finding of the present study was the identification of opposite patterns of DMN homogeneity alterations within the precuneus between adolescents with higher and lower sleep symptom burden. As a core hub of the posterior DMN, the precuneus plays an important role in self-referential processing, episodic memory retrieval, and internally directed cognition (32, 33). Increased NH in the precuneus among patients with lower sleep symptom burden is generally consistent with previous depression studies in which sleep-related differences were not specifically considered. For example, Guo et al. (34) found in a study of treatment-naïve adults with first-episode depression that patients had increased NH in the right posterior cingulate and precuneus compared to healthy controls (34), which may reflect enhanced self-related information processing in depression. In contrast, the MDD-S group exhibited reduced NH in the precuneus, which is highly consistent in direction with the findings by Tao et al. (13) of generally weakened internal functional connectivity within the DMN in depression comorbid with sleep disturbances (13), as well as the overall decline in network efficiency observed by Chen et al. in patients with chronic sleep disturbances and depression (35). These findings collectively suggest that sleep symptom burden may be associated with different patterns of DMN synchronization alterations in depression. To our knowledge, opposite-direction precuneus alterations associated with different levels of sleep symptoms have not been directly reported in previous NH studies of depression. One possible explanation is that sleep-related differences were not adequately considered in previous depression studies, potentially obscuring distinct DMN synchronization patterns associated with sleep symptoms. If patients with different levels of sleep symptoms are analyzed together, opposite-direction alterations may partially offset each other at the group level, potentially contributing to inconsistent findings across studies. Notably, the opposite direction of precuneus alterations observed between patient subgroups suggests that accounting for sleep symptom burden may help clarify some of the previously inconsistent DMN findings in depression, although this interpretation remains preliminary given the cross-sectional design. Therefore, the present findings may provide an additional perspective for interpreting previously inconsistent DMN results in depression. At the mechanistic level, the reduced network synchronization consistency in the precuneus of patients with sleep disturbances may be related to sleep deprivation–associated disruptions in intrinsic neural synchronization and functional network integrity (36, 37). Previous PET and functional connectivity studies have reported reduced precuneus metabolism and weakened functional connectivity following sleep deprivation (38, 39), which may provide physiological support for the present findings. In contrast, increased NH in the precuneus among patients with lower sleep symptom burden may reflect enhanced internal synchronization within posterior DMN regions. Notably, previous neuroimaging studies have suggested that sleep deprivation and insomnia are associated with altered precuneus activity and disrupted DMN connectivity, particularly involving self-referential processing and internal mentation (40, 41). Therefore, the opposite-direction precuneus alterations observed in the present study may be associated with different patterns of DMN synchronization related to sleep symptom burden in adolescent MDD.

Regarding sleep-related group differences, this study found that the MDD-S group exhibited increased NH in the left anterior cingulate cortex (ACC), left posterior cingulate cortex (PCC), and left angular gyrus, as well as decreased NH in the right middle temporal gyrus (MTG). Increased NH in the cingulate and angular gyri is generally consistent with the increased network synchrony reported by Chen et al. (35). The present findings provide additional observations at the level of individual DMN nodes: the anterior cingulate (emotional regulation), posterior cingulate (self-referential processing), and angular gyrus (episodic memory) all exhibited increased NH, which may tentatively suggest that patients with MDD-S exhibit relatively increased local synchronization within these regions, possibly reflecting altered neural coordination associated with sleep symptom burden. However, such interpretations remain speculative without direct measures of neural effort or compensatory processes. In contrast, Tao et al. (13) found a general reduction in intra-DMN functional connectivity in adult patients (13), differing from the findings of this study. One possible explanation for this discrepancy may involve developmental differences between adolescent and adult samples. Tao et al.’s sample consisted of adults, whereas this study involved adolescents (aged 11–18). During adolescence, the DMN remains under active developmental refinement and exhibits substantial neural plasticity (42). Sleep disturbances during adolescence may therefore be associated with altered internal DMN synchronization patterns that differ from those observed in adults. If replicated in future studies, these findings may indicate that developmental stage contributes to differences in DMN synchronization patterns associated with sleep symptoms in depression. Regarding the middle temporal gyrus (MTG), the reduced NH in the right MTG of the MDD-S group in this study is consistent with the findings of Cui et al. (28) in major depressive disorder (MDD) with depressive symptoms (28). However, unlike the findings of Yan et al. (43), who reported that reduced MTG NH in patients with depressive symptoms was associated with anhedonia scores (43), this study did not find such an association. This discrepancy may be related to differences in symptom dimensions examined across studies. Yan et al.’s study was based on a melancholic/non-melancholic classification, whereas this study was based on sleep disturbances symptom classification. Because anhedonia represents a core depressive symptom dimension, MTG alterations reported in previous studies may be more strongly associated with reward-related dysfunction than with sleep symptoms. Reduced MTG synchronization in the present study may reflect a relatively nonspecific depression-related alteration rather than a sleep-related effect. This interpretation is also broadly consistent with the trend toward reduced MTG NH observed in the group with lower sleep symptom burden.

It should also be noted that the present study focused on network homogeneity (NH), which quantifies voxel-wise synchronization within a predefined network rather than overall network activity. Therefore, the primary aim was to characterize local coherence patterns within the DMN rather than global DMN activation. Future studies combining NH with complementary measures, such as whole-network functional connectivity, amplitude-based metrics, or network-level activity indices, may provide a more comprehensive understanding of sleep-related alterations in adolescent MDD.

Notably, no significant associations were identified between NH values and clinical symptom severity. NH values in all regions of interest showed no significant correlation with either the total HAMD score or the sleep subscale score (**Supplementary Table 1**). Although these findings suggest that NH alterations may partially reflect relatively stable network-level characteristics rather than state-dependent symptom fluctuations, they should be interpreted with caution because the present analyses were exploratory and may have been influenced by the restricted range of symptom severity and sample characteristics. First, static NH measures may reflect relatively stable characteristics of network organization rather than symptom fluctuations that vary with clinical severity. This is similar to the findings of Gao et al. (30) in their study on the homogeneity of the dorsal attention network in depression, suggesting that network homogeneity alterations may represent relatively stable neural characteristics of depression rather than direct indicators of symptom severity (30). Second, sleep-related group differences may reflect distinct neural patterns that are not necessarily linearly associated with symptom severity. Intergroup differences in NH values may be associated with distinct patterns of DMN synchronization related to the presence versus absence of prominent sleep symptoms, although no significant linear correlation was found between NH values and sleep factor scores. This interpretation is also limited by the use of HAMD sleep items rather than objective sleep measures or a formal insomnia diagnosis. Therefore, variability in sleep scores within the higher sleep symptom burden group may not be sufficient to produce detectable linear changes in NH values. Some studies have also noted that static functional connectivity measures are often uncorrelated with clinical scores, whereas dynamic measures show correlations (44), further supporting the possibility that static network measures capture relatively stable neural characteristics. Third, sample characteristics may have reduced the ability to detect significant correlations. The sample consisted of adolescents with first-episode, untreated MDD and relatively short illness duration (<12 months). Clinical symptoms and neural alterations may still be undergoing developmental changes during the early stage of illness, and the relationship between NH values and clinical scores may not yet have established a stable linear pattern. In addition, the association between clinical symptoms and NH alterations may be influenced by multiple psychological, behavioral, and developmental factors that were not assessed in the present study.

Exploratory SVM analyses demonstrated modest discriminative performance for group differentiation. SVM classification results showed an accuracy of 68.35% for distinguishing MDD-S from HC, 68.22% for distinguishing MDD-NS from HC, and only 58.02% for distinguishing MDD-S from MDD-NS, indicating limited ability to distinguish groups with different levels of sleep symptom burden. Because the SVM analyses were exploratory in nature and only overall classification accuracy was retained, additional classification metrics such as sensitivity, specificity, balanced accuracy, and area under the curve (AUC) were unavailable for retrospective evaluation. Therefore, the present classification results should be interpreted cautiously. Previous studies using adult samples or multimodal approaches have reported relatively higher classification performance (16, 35, 45). Several factors may account for the modest performance observed in the current study: first, the two depressive groups likely share substantial core depressive pathology, resulting in relatively subtle neuroimaging differences; second, substantial individual variability within the adolescent sample may increase classification difficulty; and third, sleep grouping based on symptom measures rather than objective sleep assessments may have reduced subgroup separability. Therefore, future studies could integrate objective sleep monitoring (e.g., polysomnography), dynamic imaging metrics, and multimodal data to improve the characterization of sleep-related neural alterations.

Several limitations should be considered when interpreting the present findings. First, although age was included as a covariate in all analyses, the healthy control group was slightly older than the MDD groups. Given that adolescence is a period of rapid brain network maturation, including ongoing development of the DMN, residual developmental confounding cannot be completely excluded despite statistical adjustment. Future studies with age-matched samples and longitudinal designs are warranted. Second, the cross-sectional design precludes causal inference regarding the relationship between sleep symptom burden and DMN homogeneity alterations. Third, sleep-burden grouping was based on the summed HAMD sleep-factor score rather than a formal insomnia diagnosis or objective sleep assessments such as polysomnography. In addition, the three HAMD sleep items (difficulty falling asleep, middle insomnia, and early morning awakening) were not analyzed separately and may reflect partially distinct neurobiological mechanisms. Although the two MDD subgroups did not differ significantly in non-sleep HAMD scores (**Table 1**), residual confounding by unmeasured aspects of depressive symptomatology cannot be completely excluded. Future studies should investigate specific sleep dimensions using larger samples and more comprehensive sleep assessments. Fourth, healthy controls were not systematically assessed using dedicated sleep questionnaires (e.g., the Pittsburgh Sleep Quality Index [PSQI]), insomnia scales, or objective sleep measures. Although all healthy controls were screened through clinical interviews and were free of psychiatric disorders, undetected sleep disturbances cannot be completely ruled out. Consequently, comparisons between the MDD subgroups and healthy controls may reflect a combination of diagnostic effects, sleep-burden effects, and unmeasured variability in sleep status within the control group. Future studies should incorporate comprehensive sleep assessments in both patient and control groups to better disentangle these effects. Fifth, no significant correlations were observed between NH values and clinical symptom measures. Although this finding may suggest that NH alterations reflect relatively stable network-level characteristics rather than state-dependent symptom fluctuations, the correlation analyses were exploratory and should be interpreted with caution. The restricted range of symptom severity, relatively short illness duration, and developmental characteristics of the adolescent sample may have limited the ability to detect significant brain–symptom associations. In addition, although all patients met the inclusion criterion of illness duration shorter than 12 months, detailed illness-duration data were unavailable for retrospective reporting. Therefore, the potential influence of illness duration on NH alterations could not be formally evaluated. Finally, although the sample size was relatively large for a single-center adolescent rs-fMRI study, it still limited further exploration of clinical heterogeneity and the exploratory SVM analyses demonstrated only modest classification performance. These findings suggest that NH alterations alone are unlikely to serve as a robust individual-level biomarker. Future studies incorporating multimodal neuroimaging, objective sleep measures, and larger longitudinal cohorts are needed to validate and extend the present findings.

## 5 Conclusion

In conclusion, this study revealed distinct DMN homogeneity profiles in adolescents with MDD and different levels of sleep symptom burden, characterized by opposite-direction alterations in the precuneus. Although exploratory SVM analyses suggested that NH features yielded modest accuracy in differentiating patients from healthy controls (approximately 68% in both comparisons), these findings should be interpreted with caution given the exploratory nature of the classification analyses and the limited performance in distinguishing patient subgroups (58.02%). These preliminary findings suggest that sleep symptom burden may be associated with neurobiological heterogeneity in adolescent MDD and may represent an important clinical characteristic worthy of further investigation. However, replication in larger samples incorporating objective sleep assessments and longitudinal designs is required before firm conclusions regarding sleep-related stratification can be drawn.

## Data Availability

The raw data supporting the conclusions of this article will be made available by the authors, without undue reservation.
