# Supplementary material for: Association of sleep symptom burden with default mode network homogeneity in first-episode drug-naïve adolescents with major depressive disorder
Source: Front Psychiatry. 2026 Aug 6;17:1886224. doi: 10.3389/fpsyt.2026.1886224 (PMC13492238; doi:10.3389/fpsyt.2026.1886224)
Supplement: Supplementary file 1 [file DataSheet1.docx]

| Cluster location | Group | Clinical variable | ρ (Spearman) | p-value |  |
| --- | --- | --- | --- | --- | --- |
| Right Precuneus | MDD-S | HAMD total | -0.084 | 0.444 | |
| Right Precuneus | MDD-S | Sleep factor | 0.101 | 0.356 | |
| Left Precuneus | MDD-S | HAMD total | 0.112 | 0.303 | |
| Left Precuneus | MDD-S | Sleep factor | -0.010 | 0.925 | |
| Left Anterior Cingulum | MDD-S | HAMD total | 0.041 | 0.710 | |
| Left Anterior Cingulum | MDD-S | Sleep factor | -0.254 | 0.060 | |
| Left Posterior Cingulum | MDD-S | HAMD total | 0.080 | 0.465 | |
| Left Posterior Cingulum | MDD-S | Sleep factor | -0.008 | 0.944 | |
| Left Angular Gyrus | MDD-S | HAMD total | 0.158 | 0.147 | |
| Left Angular Gyrus | MDD-S | Sleep factor | 0.108 | 0.324 | |
| Right Middle Temporal Gyrus | MDD-S | HAMD total | -0.147 | 0.175 | |
| Right Middle Temporal Gyrus | MDD-S | Sleep factor | -0.151 | 0.167 | |

**Supplementary Table S1. Correlations between NH values extracted from significant clusters and clinical variables (HAMD total score and sleep factor score).** All correlations were non-significant (p > 0.05).

**Supplementary Figure S1. Group ICA-derived DMN component.** The ICA-derived component showed spatial correspondence with the canonical DMN, including the precuneus, posterior cingulate cortex (PCC), medial prefrontal cortex (mPFC), and bilateral angular gyri (AG).
